# Trends in Self-Reported Responses to Nutrition Facts Labels Before and After Nutrition Labeling Policy Implementation: A Comparison of Adults in the United States and Mexico

**DOI:** 10.1016/j.cdnut.2026.107661

**Published:** 2026-02-22

**Authors:** James F Thrasher, Victor Villalobos, Dai Fang, Alejandra Jáuregui, Alejandra Contreras-Manzano, Rachel E Davis, Lana Vanderlee, Christine M White, Rachel B Acton, James W Hardin, Edward A Frongillo, Simón Barquera, David Hammond

**Affiliations:** 1 Department of Health Promotion, Education & Behavior, Arnold School of Public Health, University of South Carolina, Columbia, SC, United States; 2 Center for Health and Nutrition Research, Instituto Nacional de Salud Pública, Morelos, Cuernavaca, Mexico; 3 Nutrition and Mental Health Department, Pan American Health Organization, Washington, DC, United States; 4 Department of Epidemiology and Biostatistics, Arnold School of Public Health, University of South Carolina, Columbia, SC, United States; 5 National Council for Humanities, Science and Technology, Mexico City, Mexico; 6 École de nutrition, Faculté des sciences de l’agriculture et de l’alimentation (FSAA), Centre NUTRISS, Université Laval, Québec, QC, Canada; 7 Centre Nutrition, santé et société (NUTRISS), INAF, Université Laval, Québec, QC, Canada; 8 School of Public Health Sciences, University of Waterloo, Waterloo, ON, Canada

**Keywords:** nutrition labeling, nutrition policy, policy evaluation, health communication, consumer perceptions

## Abstract

**Background:**

In 2020–2021, both Mexico and the United States implemented similar, newly formatted nutrition facts labels (NFLs). Mexico simultaneously implemented front-of-package warning labels (FoPWLs), which emphasize high amounts of less healthy nutrients described in NFLs (e.g., calories, sugar, salt, trans fat, and saturated fat) to enhance consumer understanding of nutrition information.

**Objectives:**

To evaluate these policies by comparing pre- and postpolicy trends in self-reported responses to NFLs.

**Methods:**

Annual surveys from the adult International Food Policy Study (2018–2023) were analyzed for Mexico (*n* = 24,832) and the United States (*n* = 25,464). Outcomes included reported ease of finding nutrition information in grocery stores and awareness, use, and understanding of NFLs (all measured on 1–5 Likert scales). A difference-in-differences method using adjusted and weighted linear regression models compared cross-country differences in trends for these outcomes over the transition (2019–2020), early (2019–2021), mid (2019–2022), and late (2019–2023) postimplementation periods relative to prepolicy trends (2018–2019).

**Results:**

Trends over the transition period (compared with prepolicy) were stable within and across countries. For all outcomes, trends up to the early implementation period (compared with prepolicy) were more positive in Mexico than in the United States (i.e., ease of finding nutrition information *B* = 0.195, *P* = 0.003; awareness *B* = 0.220, *P* < 0.001; understanding *B* = 0.332, *P* < 0.001; and use *B* = 0.211, *P* = 0.006), driven by both increases in Mexico (i.e., ease of finding *B* = 0.126, *P* = 0.008; awareness *B* = 0.132, *P* = 0.001; understanding *B* = 0.198, *P* < 0.001; and use *B* = 0.087, *P* = not significant [ns]) and decreases in the United States (i.e., ease of finding information *B* = ns; awareness *B* = −0.088, *P* = −0.049; understanding *B* = −0.134, *P* = 0.002; and use *B* = −0.124, *P* = 0.025). When evaluating mid and late postimplementation periods, contrasts favored Mexico over the United States for all outcomes, except NFL use, which did not differ within or across countries.

**Conclusions:**

Newly formatted NFLs in the United States did not increase awareness, understanding, or use of NFLs, particularly when compared with Mexico’s new NFLs that were accompanied by FoPWLs. Further research should determine the labeling effects on eating behaviors.

## Introduction

Food labeling is a cost-effective, population-level intervention to communicate nutrition information to consumers and, thereby, support informed and healthier food choices. Consumers are exposed to food labels at critical points of product selection and consumption, frequently use labels [1,2], and trust labels more than other nutrition information [[3], [4], [5]]. Food label use is associated with improved diet quality [6,7], including lower intake of fat [[8], [9], [10]], sugar-sweetened beverages [11,12], and total energy [11,12]. Countries often require that packaged foods include nutrient declarations in the form of nutrition facts labels (NFLs) on the back or side of food packaging. However, many consumers struggle to understand NFLs [2,[13], [14], [15], [16]], including in functional tasks [[17], [18], [19], [20]] or when using calculations required to interpret NFLs [17,[21], [22], [23]]. In response, some countries have updated NFLs to improve their effectiveness.

**FIGURE 1:**
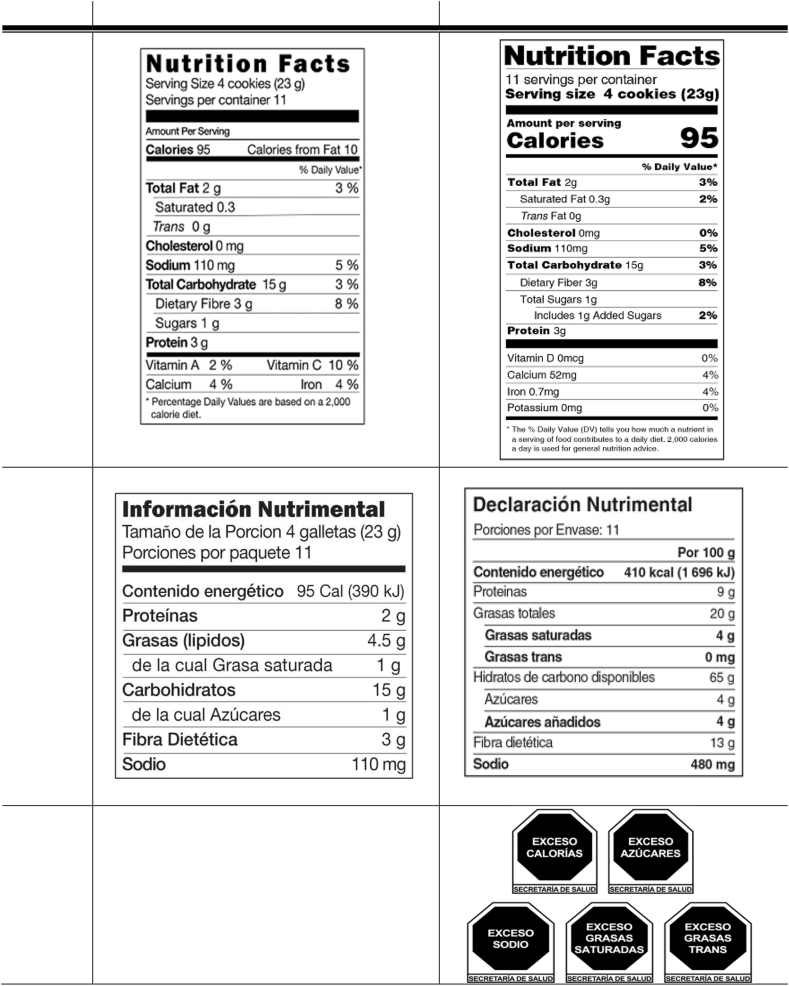
Nutrition labeling pre- and post-2020/-2021 policy implementation in Mexico and the United States. Column 2 header: Pre-2020/2021 policy implementation. Column 3 header: Post-2020/2021 policy implementation; Row 2 label: United States: nutrition facts labels; Row 3 label: Mexico: nutrition facts labels.^1^ Row 4 label: Mexico: front-of-package warning labels.^2^ Footnotes: 1. Translations: Información nutrimental = nutrient information; Declaración nutrimental = nutrient declaration; tamaño de porción = portion size; porciones por paquete = portions per pack; porciones por envase = portions per container; contenido energético = calories; proteínas = proteins; grasas = fats; grasa saturada = saturated fat; grasas trans = transfats; carbohidrátas = carbohydrates; hidratos de carbono disponibles = available carbohydrates; azúcares = sugars; azucares añadidos = added sugars; fibra dietética = dietary fiber; sodio = salt; 2. Translations: Exceso = excess; Secretaría de Salud = Ministry of Health (other terms shown in footnote 1).

Since 1990, the United States has required NFLs on all prepackaged foods using a standardized format for listing energy (i.e., calories), other contents, and a percent daily value for key nutrients [24]. More than 60% of the United States consumers have reported using NFLs [2,8], although use has declined over time [25]. Between January 2020 and December 2021, the United States phased in updated NFLs [26] with more salient serving sizes and calorie information (i.e., bolded, larger font), as well as new information about added sugars and changes to displays of micronutrients for standardized serving sizes (Figure 1) [27]. Preimplementation experiments found both positive [27,28] and no effect [29] of these NFL changes. To our knowledge, no population-level evaluations of this policy have been published.

In Mexico, NFLs have been mandatory on the back of all packaged foods since 1996 [30], with format and content similar to the United States, although without mandating standard portion sizes. A March 2020 law mandated that by April 2021, NFLs had to use bolding (without bigger font) to highlight key nutrients of concern: saturated fat, trans fat, added sugars, and sodium (Figure 1). The updated Mexican NFLs also required mandatory nutrition information to be presented in standardized units (i.e., per 100 g/mL). Declaration of vitamin or mineral content (other than sodium) is optional, except for foods and nonalcoholic beverages whose composition is modified or fortified [31].

To further improve consumer understanding of nutrition information, Mexico’s 2020 labeling law also mandated the display of front-of-package warning labels (FoPWLs) on unhealthy foods and beverages. Implementation of the labels began in October 2020 and was required by June 2021. Packaged products that exceed thresholds for “excess” amounts of calories, added sugars, saturated fats, trans fats, and/or sodium (Figure 1) must now prominently display octagonal, black, and white “stop sign” symbols indicating that the product is “high in” those nutrients of concern [32]. Packages may display up to 5 octagons, depending on the number of thresholds triggered. Additionally, rectangular legends are also required on foods that contain non-nutritive sweeteners or caffeine. Qualitative and experimental studies suggest that FoPWLs are more noticeable and more easily understood than NFLs, including among Mexican and United States adults [33,34]. Furthermore, the location of FoPWLs on the front of packages and their ease of interpretation allows for rapid comparison of products within and between the same category at the point of selection—a process that is much more time consuming and complex when comparing NFLs [35].

FoPWLs are designed to allow relatively easy and fast decision making. This type of rapid, heuristic reasoning can generate a “spotlight” effect that leads to more deliberative decision making by promoting greater scrutiny of information related to the source [36]. In this regard, FoPWLs may not only raise concerns about nutritional content, but they may also promote greater awareness, use, and understanding of NFLs, which, on their own, require more effortful processing than FoPWLs. As such, due to the standardization of NFL information and the presence of FoPWLs accompanying the NFLs in Mexico, we hypothesized that pre-/postpolicy changes in awareness, use, and understanding of NFLs would be greater in Mexico than in the United States.

The current study extends prior research from the International Food Policy Study (IFPS) that reported on within-country changes in adults’ awareness, understanding, and use of labels from 2018 to 2020 in the United States, Mexico, Australia, the United Kingdom, and Canada [37]. We focus on self-reported awareness, understanding, and use of NFLs, given that these outcomes are associated with changes in nutrition knowledge and behavior [[38], [39], [40]]. Previous IFPS research stratified analyses by country [37] and found that, relative to 2018, awareness of NFLs in the United States decreased through 2020, and no change was found in NFL use or understanding, whereas in Mexico, NFL awareness, understanding, and use increased or did not change, depending on the contrast with 2018 data. However, this prior study did not compare prepolicy trends (i.e., from 2018 to 2019, or the secular trend in the absence of policy change) with postpolicy trends or test whether these trends were significantly different across countries. Furthermore, the 2020 survey—the most recent data analyzed in the prior study—was administered during the transition period before full NFL policy implementation in 2021 for both Mexico and the United States. Finally, 2020 data are from the first year after the onset of the COVID-19 pandemic, which significantly altered food purchasing and consumption [41,42]; thus, evaluation of later data is needed to better capture policy effects. As such, the current study used a difference-in-differences method to assess differences in trends from the prepolicy period (i.e., 2018–2019) to the postpolicy period, with contrasts across trends covering the transition period (2019–2020), as well as early (2019–2021), mid (2019–2022), and late (2019–2023) postimplementation periods, comparing the United States and Mexican adults.

## Methods

### Sample

Data were analyzed from the IFPS’s repeat cross-sectional online survey of adults aged 18–100, conducted annually in November and December, allowing for a difference-in-difference assessment of changes across countries over time. Our analyses were restricted to Mexico and United States surveys from 2018 to 2023. The IFPS sample size was not determined from power analysis for the current study but for the IFPSs’ overall objective of detecting generally meaningful changes in consumer perceptions and behaviors over time. Samples were recruited through the Nielsen Consumer Insights Global Panel using target quotas for age, sex, and educational attainment groups to approximate each country’s population distribution across these strata. Online surveys were self-administered in English or Spanish in the United States and in Spanish in Mexico, and compensation involved usual panel incentives (e.g., points-based rewards). Cooperation rates for eligible participants who accessed the survey link ranged from 49.8% (2020) to 71.0% (2018) in Mexico (2019 = 60.1%; 2021 = 67.7%; 2022 = 64.6%; and 2023 = 64.0%) and 49.9% (2019) to 69.2% (2021) in the United States (2018 = 68.0%; 2020 = 63.3%; 2022 = 67.9%; and 2023 = 66.4%). Study protocols were reviewed by and received ethics clearance through a University of Waterloo Research Ethics Committee (Research Ethics Board #30829), the Institutional Review Board of the University of South Carolina, and the Ethics Committee of the National Institute of Public Health of Mexico (Instituto Nacional de Salud Pública).

### Measures

#### Main outcomes

Participants reported their perception of the ease of finding nutrition information in grocery stores (i.e., “In your opinion, is nutrition information easy or hard to find in grocery stores? *1*) Very hard to find, *2*) Hard to find, *3*) Neither hard nor easy, *4*) Easy to find, and *5*) Very easy to find”). A series of questions about NFLs involved showing an image of the NFL from the participants’ country (displaying consistent nutrient values across countries, with the updated NFL shown in the United States starting in 2020 and in Mexico starting in 2022; Figure 1). Participants reported their awareness of NFLs (i.e., “How often have you seen this type of food label on packages or in stores? *1*) Never, *2*) Rarely, *3*) Sometimes, *4*) Often, and *5*) All the time”); understanding of NFLs (i.e., “Do you find this information…: *1*) Very hard to understand, *2*) Hard to understand, *3*) Neither hard nor easy, *4*) Easy to understand, and *5*) Very easy to understand”); and, among those who reported any NFL awareness (≥ “Rarely” seen NFLs), the frequency of NFL use (i.e., “How often do you use this type of food label when deciding to buy a food product?: *1*) Never, *2*) Rarely, *3*) Sometimes, *4*) Often, and *5*) All the time”). Those who reported “never” for NFL awareness were recoded to “never” for NFL use.

#### Covariates

To account for potential differences in opportunities for exposure to labels, participants reported their role in household food purchasing (i.e., “How much of the food shopping do you do in your household?”), with responses dichotomized (i.e., “Most” or “Shared equally” compared with “Some, but less than others,” or “None”). Because having children may influence purchase behaviors, participants reported the number of children <18 y who lived in their households (recoded to none compared with any children). Age was evaluated as continuous, biological sex at birth was reported as male or female, and ethnicity was recoded to indicate either majority (i.e., the United States: non-Latino White; Mexico: nonindigenous) or minority (i.e., the United States: all other racial and ethnic groups; Mexico: indigenous) ethnicity, which allowed for harmonization across countries. Educational attainment was recoded into 3 levels for the United States (i.e., low = high school or lower; medium = associate degree; and high = university degree or higher) and Mexico (i.e., low = none, preschool, primary, secondary, high school, or baccalaureate, normal basic; medium = technical or commercial studies with completed primary, technical, or commercial studies with completed secondary, technical, or commercial studies with completed high school; and high = bachelor’s degree or higher). Perceived income adequacy was also assessed (“Thinking about your total monthly income, how difficult or easy is it for you to make ends meet?: Very difficult, Difficult, Neither, Easy, and Very easy”), as a validated indicator of relative social position that facilitates cross-country comparisons [43].

### Analysis

Unadjusted and adjusted linear regression models were estimated for each main outcome, with adjusted models including all covariates (see above). The difference-in-differences method involved evaluating whether countries differed from each other in the difference between the prepolicy trend from 2018 to 2019 (i.e., what would have been the trend in the postimplementation period had labeling regulations not changed) and the postpolicy trend, which we estimated for the initial policy transition year (2019–2020), as well as early (2019–2021), mid (2019–2022), and late (2019–2023) postpolicy implementation periods. Each postpolicy trend involved estimating the slope from 2019 to the survey year of interest, without considering data from surveys conducted in the interval between these 2 y. Coding for these contrasts aligned with our hypothesis that the postpolicy compared with prepolicy slopes would be greater in Mexico than in the United States. All analyses were adjusted for poststratification weights. Each survey year, these weights were developed using a raking algorithm based on population distributions of sex, age group, region, ethnicity, and, in the United States only, educational attainment. In Mexico, the sample of participants with low education was too small to include education in reliable weight construction. Weights were then rescaled to sum to the actual sample size for each country. All covariates were included in the adjusted models. Using postestimation commands in adjusted models for each outcome, we plotted the mean predicted probabilities in each country for each survey year. To assess the difference-in-difference assumption of prepolicy parallel trends in outcomes, we limited data to the 2018 and 2019 surveys and tested interactions between country and the prepolicy time trend; results indicated no difference in trends over the prepolicy period (*P* = 0.32–0.75), except for NFL understanding (*P* = 0.02).

We also ran sensitivity analyses to compare our linear regression results with ordered logistic regression (using original responses). Results were consistent in terms of the valence, confidence, and interpretation as those that we report here for linear regression (Supplemental Table 1). Missing data due to providing “don’t know” or “refused” responses for outcomes were relatively low (range: 0.5%–1.4%), as was additional missingness for any independent variables used in the adjusted analyses (range: 2.9%–3.6%). Results from multiple imputation models with chained equations were also consistent with the results reported here (Supplemental Table 2). Analyses were conducted using SAS, version 9.4 (https://www.sas.com/en_us/home.html).

## Results

About half of the participants were female in each country (Table 1). In Mexico, the sample was younger (mean: 40.3 y old compared with 47.0 y old in the United States) and had a higher percentage of participants with high educational attainment (64.7% compared with 33.8%), had children at home (49.8% compared with 28.5%), and reported being the primary household shopper (73.2% compared with 69.4%).

**TABLE 1:** Sample characteristics of adults in the United States and Mexico, 2018–2023.

Characteristics		*n*	United States		*n*	Mexico		*P* value^1^
			%/Mean (SD) unweighted	%/Mean (SD) weighted		%/Mean (SD) unweighted	%/Mean (SD) weighted	
Sex	Male	12,324	48.4	48.9	12,623	50.8%	47.8	—
	Female	13,140	51.6	51.1	12,209	49.2%	52.2	0.042
Age (y)	Mean (SD)	25,464	48 (17.0)	47 (17.1)	24,832	38 (12.9)	40.3 (14.4)	<0.001
Education	Low	9304	36.7	56.3	5221	21.1%	21.4	<0.001
	Medium	5336	21.0	9.9	3214	13.0%	13.8	—
	High	10,715	42.3	33.8	16,358	66.0%	64.7	—
Perceived Income Adequacy	Very difficult	2274	9.0	10.2	2687	10.9%	11.9	<0.001
	Difficult	4778	19.0	20.0	7676	31.1%	32.0	—
	Neither easy nor difficult	8100	32.2	33.7	9744	39.5%	39.0	—
	Easy	5777	22.9	20.9	3564	14.5%	13.4	—
	Very easy	4252	16.9	15.2	979	4.0%	3.7	—
Children in household	None	17,936	70.6	71.5	11,570	46.6%	50.2	<0.001
	One or more	7476	29.4	28.5	13,233	53.4%	49.8	—
Ethnicity	Majority	18,029	71.3	63.3	20,145	82.9%	80.1	<0.001
	Minority	7241	28.7	36.7	4170	17.1%	19.9	—
Household Shopping	Primary shopper	18,025	71.0	69.4	17,956	72.4%	73.2	<0.001
	Not primary shopper	7346	29.0	30.6	6850	27.6%	26.8	—
Survey year	2018	4640	18.2	18.2	4135	16.7	16.7	<0.001
	2019	4183	16.4	16.4	4314	17.4	17.4	—
	2020	4622	18.2	18.2	4284	17.3	17.3	—
	2021	4093	16.1	16.1	4172	16.8	16.8	—
	2022	3985	15.6	15.6	4153	16.7	16.7	—
	2023	3941	15.5	15.5	3774	15.2	15.2	—

1 *P* value for survey-weighted linear regression (age only) or Rao–Scott chi-square from survey-weighted tests comparing the United States and Mexico sample.

### Ease of finding nutrition information in grocery stores

**FIGURE 2:**
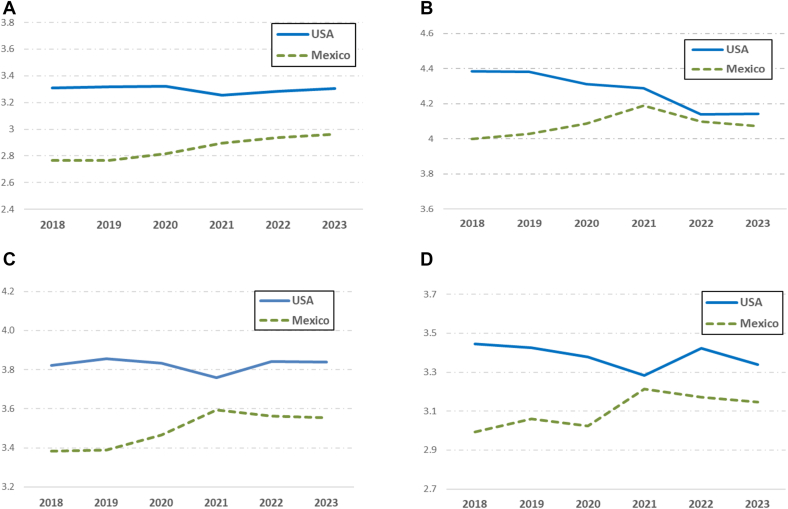
Trends in outcomes∗ related to nutrition facts labeling (NFL) policy among adults in Mexico and the United States, 2018–2023. (Upper left panel, A) Ease of finding nutrition information in grocery stores; (upper right panel, B) extent of awareness of NFLs; (lower left panel, C) ease of understanding NFLs; (lower right panel, D) frequency of using NFLs. Footnote: ∗Responses on 1–5 point Likert scale (i.e., A and C: 1—very hard to 5—very easy; B and D: 1 = never to 5 = all the time; point estimates are derived from adjusted and weighted model.

Trends in the ease of finding nutrition information in stores during the policy transition period (2019–2020) relative to the prepolicy period (2018–2019) were not different within Mexico, within the United States, or when comparing the 2 countries (Table 2 and Figure 2A). When evaluating differences in trends for early policy implementation compared with prepolicy (2019–2021 compared with 2018–2019); however, the ease of finding nutrition information increased in Mexico (*B*_adj_: 0.126; 95% confidence interval [CI]: 0.032, 0.220; *P* = 0.008), and this increase was stronger in Mexico than in the United States (*B*_adj_: 0.195; 95% CI: 0.067, 0.323; *P* = 0.003), where no change was observed. This same pattern of results was found when comparing baseline trends with the mid and late postimplementation periods (i.e., 2019–2022 and 2019–2023), where the ease of finding nutrition information increased in Mexico (*B*_adj_: 0.167; 95% CI: 0.074, 0.261; *P* < 0.001; *B*_adj_: 0.192; 95% CI: 0.098, 0.286; *P* < 0.001, respectively) and between-country contrasts favored Mexico over the United States (*B*_adj_: 0.204; 95% CI: 0.076, 0.322; *P* = 0.003; *B*_adj_: 0.208; 95% CI: 0.080, 0.336; *P* = 0.001).

**TABLE 2:** Post- vs. prepolicy trends for the salience of nutrition information in stores and awareness, understanding, and use of NFLs: Mexico, the United States, and Mexico vs. United States, 2018–2023.

Period contrasts	Country contrasts	B	(95% CI)	*P* value	*B*_adj_^1^	(95% CI)	*P* value
**Ease of finding nutrition informationin stores**							
Initial policy transition (2019–2020) vs. prepolicy period (2018–2019)	Mexico (within country)	0.012	(−0.084, 0.109)	0.800	0.048	(−0.046, 0.143)	0.316
	United States (within country)	0.000	(−0.088, 0.089)	0.991	0.001	(−0.086, 0.088)	0.985
	Mexico vs. United States	0.012	(−0.119, 0.143)	0.858	0.048	(−0.081, 0.176)	0.468
Early policy implementation (2019–2021) vs. prepolicy period (2018–2019)	Mexico (within country)	0.127	(0.031, 0.223)	0.009	0.126	(0.032, 0.220)	0.008
	United States (within country)	−0.033	(−0.122, 0.055)	0.459	−0.069	(−0.156, 0.018)	0.120
	Mexico vs. United States	0.161	(0.030, 0.291)	0.016	0.195	(0.067, 0.322)	0.003
Mid policy implementation (2019––2022) vs. prepolicy period (2018–2019)	Mexico (within country)	0.151	(0.055, 0.246)	0.002	0.167	(0.074, 0.261)	<0.001
	United States (within country)	−0.044	(−0.134, 0.046)	0.334	−0.037	(−0.125, 0.051)	0.411
	Mexico vs. United States	0.195	(0.064, 0.327)	0.004	0.204	(0.076, 0.333)	0.002
Late policy implementation (2019–2023) vs. prepolicy period (2018–2019)	Mexico (within country)	0.189	(0.093, 0.285)	<0.001	0.192	(0.098, 0.286)	<0.001
	United States (within country)	−0.017	(−0.106, 0.072)	0.708	−0.016	(−0.103, 0.071)	0.720
	Mexico vs. United States	0.206	(0.076, 0.337)	0.002	0.208	(0.080, 0.336)	0.001
**Extent of awareness of NFLs**							
Initial policy transition (2019–2020) vs. prepolicy period (2018–2019)	Mexico (within country)	0.038	(−0.041, 0.117)	0.348	0.031	(−0.049, 0.111)	0.447
	United States (within country)	−0.069	(−0.158, 0.019)	0.124	−0.065	(−0.151, 0.021)	0.140
	Mexico vs. United States	0.107	(−0.011, 0.226)	0.076	0.096	(−0.022, 0.214)	0.110
Early policy implementation (2019–2021) vs. prepolicy period (2018–2019)	Mexico (within country)	0.144	(0.065, 0.224)	<0.001	0.132	(0.052, 0.212)	0.001
	United States (within country)	−0.071	(−0.161, 0.019)	0.120	−0.088	(−0.176, −0.001)	0.049
	Mexico vs. United States	0.215	(0.096, 0.335)	<0.001	0.220	(0.102, 0.339)	<0.001
Mid policy implementation (2019–2022) vs. prepolicy period (2018-2019)	Mexico (within country)	0.052	(−0.027, 0.132)	0.196	0.043	(−0.037, 0.123)	0.294
	United States (within country)	−0.236	(−0.328, −0.145)	<0.001	−0.238	(−0.327, −0.149)	<0.001
	Mexico vs. United States	0.289	(0.168, 0.410)	<0.001	0.281	(0.161, 0.401)	<0.001
Late policy implementation (2019–2023) vs. prepolicy period (2018–2019)	Mexico (within country)	0.025	(−0.055, 0.105)	0.540	0.016	(−0.065, 0.096)	0.703
	United States (within country)	−0.230	(−0.320, −0.139)	<0.001	−0.235	(−0.324, −0.147)	<0.001
	Mexico vs. United States	0.255	(0.134, 0.376)	<0.001	0.251	(0.131, 0.371)	<0.001
**Ease of understanding NFLs**							
Initial policy transition (2019–2020) vs. prepolicy period (2018–2019)	Mexico (within country)	0.038	(−0.059, 0.135)	0.440	0.068	(−0.028, 0.163)	0.164
	United States (within country)	−0.063	(−0.150, 0.024)	0.156	−0.060	(−0.145, 0.026)	0.173
	Mexico vs. United States	0.101	(−0.029, 0.232)	0.128	0.127	(−0.001, 0.256)	0.052
Early policy implementation (2019–2021) vs. prepolicy period (2018–2019)	Mexico (within country)	0.203	(0.105, 0.300)	<0.001	0.198	(0.103, 0.294)	<0.001
	United States (within country)	−0.100	(−0.188, −0.012)	0.026	−0.134	(−0.220, −0.048)	0.002
	Mexico vs. United States	0.302	(0.171, 0.434)	<0.001	0.332	(0.203, 0.461)	<0.001
Mid policy implementation (2019–2022) vs. prepolicy period (2018–2019)	Mexico (within country)	0.155	(0.059, 0.251)	0.002	0.166	(0.072, 0.261)	0.001
	United States (within country)	−0.059	(−0.148, 0.029)	0.190	−0.052	(−0.139, 0.035)	0.241
	Mexico vs. United States	0.214	(0.083, 0.345)	0.001	0.218	(0.090, 0.347)	0.001
Late policy implementation (2019–2023) vs. prepolicy period (2018–2019)	Mexico (within country)	0.157	(0.061, 0.254)	0.001	0.158	(0.063, 0.252)	0.001
	United States (within country)	−0.053	(−0.140, 0.035)	0.236	−0.053	(−0.139, 0.033)	0.227
	Mexico vs. United States	0.210	(0.080, 0.341)	0.002	0.211	(0.083, 0.338)	0.001
**Frequency of using NFLs**							
Initial policy transition (2019–2020) vs. prepolicy period (2018–2019)	Mexico (within country)	−0.127	(−0.234, −0.021)	0.019	−0.105	(−0.209, −0.001)	0.047
	United States (within country)	−0.017	(−0.127, 0.092)	0.758	−0.031	(−0.139, 0.077)	0.575
	Mexico vs. United States	−0.110	(−0.263, 0.042)	0.157	−0.074	(−0.224, 0.076)	0.333
Early policy implementation (2019–2021) vs. prepolicy period (2018–2019)	Mexico (within country)	0.098	(−0.008, 0.204)	0.070	0.087	(−0.017, 0.191)	0.100
	United States (within country)	−0.088	(−0.198, 0.023)	0.120	−0.124	(−0.233, −0.015)	0.025
	Mexico vs. United States	0.186	(0.033, 0.339)	0.017	0.211	(0.061, 0.362)	0.006
Mid policy implementation (2019–2022) vs. prepolicy period (2018–2019)	Mexico (within country)	0.043	(−0.062, 0.148)	0.418	0.044	(−0.058, 0.147)	0.398
	United States (within country)	0.018	(−0.093, 0.129)	0.756	0.014	(−0.095, 0.124)	0.799
	Mexico vs. United States	0.026	(−0.127, 0.178)	0.741	0.030	(−0.120, 0.180)	0.696
Late policy implementation (2019–2023) vs. prepolicy period (2018–2019)	Mexico (within country)	0.023	(−0.083, 0.129)	0.671	0.020	(−0.084, 0.123)	0.708
	United States (within country)	−0.061	(−0.171, 0.049)	0.277	−0.070	(−0.179, 0.039)	0.209
	Mexico vs. United States	0.084	(−0.069, 0.237)	0.281	0.090	(−0.061, 0.240)	0.242

Abbreviation: NFL, nutrition facts labels.

1 Adjusted for sex, age, education, income adequacy, ethnicity, having children at home, shopping role, and weights.

### Awareness of NFLs

When assessing trends in the extent of awareness of NFLs in adjusted models (Table 2 and Figure 2B), changes in the policy transition year were not large within either country relative to the prepolicy period or when comparing countries. Trends after early policy implementation relative to the prepolicy period increased within Mexico (*B*_adj_: 0.132; 95% CI: 0.052, 0.212; *P* = 0.001) and decreased within the United States (*B*_adj_: −0.088; 95% CI: −0.176, −0.001; *P* = 0.049), with a greater positive effect in Mexico relative to the contrasting trends in the United States (*B*_adj_: 0.220; 95% CI: 0.102, 0.339; *P* < 0.001). Contrasts also favored Mexico over the United States when comparing baseline trends with mid and late postimplementation periods (i.e., 2019–2022 *B*_adj_: 0.281; 95% CI: 0.161, 0.401; *P* < 0.001; 2019–2023 *B*_adj_: 0.251; 95% CI: 0.131, 0.370; *P* < 0.001), driven primarily by continued declines in awareness within the United States (*B*_adj_: −0.238; 95% CI: −0.327, −0.149; *P* < 0.001; *B*_adj_: −0.235; 95% CI: −0.334, −0.147; *P* < 0.001).

### Ease of understanding NFLs

In the adjusted model assessing trends in the ease of understanding of NFLs, differences over the policy transition period relative to the prepolicy period were not different within or between Mexico and the United States (Table 2 and Figure 2C). When evaluating differences up to the early implementation period relative to the prepolicy period (2019–2021 compared with 2018–2019), the ease of understanding nutrition information increased in Mexico (*B*_adj_: 0.198; 95% CI: 0.103, 0.294; *P* < 0.001) and decreased in the United States (*B*_adj_: −0.134; 95% CI: −0.220, −0.048; *P* = 0.002), with a relatively greater positive effect observed in Mexico compared with the United States (*B*_adj_: 0.332; 95% CI: 0.203, 0.461; *P* < 0.001). In the mid and late postpolicy implementation periods, contrasts with baseline trends were no different within the United States, remained higher within Mexico (2019–2022 *B*_adj_: 0.166; 95% CI: 0.072, 0.261; *P* < 0.001; 2019–2023 *B*_adj_: 0.158; 95% CI: 0.063, 0.261; *P* = 0.001) compared with prepolicy trends, and favored Mexico over the United States (*B*_adj_: 0.218; 95% CI: 0.090, 0.347; *P* = 0.001; *B*_adj_: 0.211; 95% CI: 0.083, 0.338; *P* = 0.001, respectively).

### Frequency of using NFLs

Reported use of NFLs in Mexico over the policy transition period (2019–2020) decreased relative to the positive trend in the prepolicy period (*B*_adj_: −0.105; 95% CI: −0.209, −0.001; *P* = 0.047), but trends were not different across these periods within the United States or when comparing Mexico with the United States (Table 2 and Figure 2D). The early policy implementation trend was no different than the prepolicy period within Mexico, relatively more negative within the United States (*B*_adj_: −0.124; 95% CI: −0.233, −0.015; *P* = 0.025), and relatively more positive in Mexico compared with in the United States (*B*_adj_: 0.211; 95% CI: 0.061, 0.362; *P* = 0.006). Contrasts with mid and late postimplementation periods were no different than baseline trends, whether examined within or across countries.

## Discussion

After full implementation, the Mexican nutrition labeling policy that revised the NFL format and adopted for the first time complementary FoPWLs appeared more effective than the United States policy that only revised the NFL format in increasing adults’ perceived ease of finding nutrition information when grocery shopping, as well as in promoting awareness, understanding, and use of NFLs. Both countries implemented changes to NFLs (e.g., making calories more prominent, requiring information about added sugars). The United States added some information (e.g., micronutrients), whereas Mexico standardized the reference units for information presented (100 g/mL) and used bold font for information about nutrients of concern. The greater relative increase in understanding and use of the revised NFLs compared with prior NFLs in Mexico was perhaps due to the standardization of units for the presentation of nutrition information, which facilitates comparison of nutrition information between and within food groups. The United States had already standardized serving sizes based on amounts typically consumed per food product category, which may also help explain the overall higher awareness, understanding, and use of NFLs in the United States than in Mexico.

The new Mexican NFLs increased the prominence of critical nutrient contents that were also highlighted in the accompanying policy of FoPWLs (e.g., salt, trans fat, saturated fat, and sugars). Except for calories, these nutrients are not prominently displayed in United States NFLs. Indeed, the FoPWLs were designed not only to help consumers easily identify packaged foods with high amounts of nutrients of concern but also to facilitate understanding and interpretation of nutrition information contained in the NFLs. As such, the FoPWLs may have generated greater attention to and use of NFLs [37], likely because they involve the prominent placement of heuristic symbols. Hence, the greater relative increases in awareness, perceived ease of understanding, and use of NFLs that we found may be due to the “spotlight” effect [36] that FoPWLs generate by prompting NFL engagement and understanding. Indeed, this supplementary labeling policy reinforces and helps interpret potential harms from packaged foods, including but going beyond calories [44].

The trends we assessed were generally stronger when contrasting the prepolicy period (2018–2019) with the early implementation period (2019–2021) than when contrasting it with the policy transition period (2019–2020). The relatively weak to null effects for the transition period within both countries may reflect effects related to the initial onset of the COVID-19 pandemic, such as reduced labeling exposure (e.g., more rapid decision making in stores, online purchasing). Also, the policy transition period was before new NFLs were required for all packaged products in Mexico (April 2021). Furthermore, although the FoPWL regulation came into effect on October 1, 2020, manufacturers had a grace period until 1 December, 2020, to put FoPWL stickers on packaging, with full implementation of FoPWLs printed on packaging required by May 2021. Products manufactured before these dates could remain on shelves for weeks or even months. Hence, results from after full implementation in 2021 provide a more adequate evaluation of the Mexican labeling policy.

Self-reported ease of finding nutrition information in stores—an outcome that presumably encompasses both NFLs and FoPWLs—continued to strengthen over the mid (2022) and late (2023) postimplementation periods in Mexico. By contrast, NFL awareness, understanding, and use showed evidence of “wear out” over the later years after implementation, similar to the trends observed with tobacco product labeling [45,46]. This may be because the spotlight effect of FoPWLs engaged consumers with NFLs soon after their implementation, and additional strategies may be needed to re-engage consumers with labeling over time (e.g., changing the format or contrasts in FoPWLs). Nevertheless, initial changes in labeling may still have led to sustained changes in the selection of healthier foods, even as NFL use waned. Prepost policy research on consumption patterns is needed to evaluate this possibility.

Trends in the United States are challenging to interpret given the observed declines in awareness, understanding, and use of NFLs for early policy implementation versus the prepolicy period, with declines in awareness continuing through 2022 and 2023. These declines occurred despite the United States Food and Drug Administration (FDA)’s information campaign to promote the new NFLs through social media, indoor and outdoor advertising, videos, and consumer-friendly downloadable educational materials [47]. Our results suggest that the changes to the NFL in the United States did not achieve the desired results, contrary to what was expected based on experimental studies [27,28], with the exception of 1 study that found no effects [29]. Experimental studies assess attention and responses under controlled conditions of “forced exposure” rather than real-life exposure to NFLs [48]. Although our study did not involve forced exposure, additional ecologically valid research from nationally representative surveys is needed to confirm our findings. We found no evidence of desirable changes within the United States over the 2020–2023 postimplementation period for any outcome we assessed. This might be because the challenges that many people face in understanding and applying the quantitative information in NFLs to make healthy food choices [2,[13], [14], [15], [16]] were unlikely to have been addressed with these minor changes. Other labeling strategies that better highlight and help consumers understand the unhealthiness of packaged foods, such as FoPWLs, are likely needed to address these concerns. Indeed, the FDA has been actively considering front-of-package labeling strategies that could help.

Our study has some limitations. Our samples came from online consumer panels, using target quotas to approximate age, sex, and educational attainment distributions in the general population. The Mexico sample, in particular, over-represented more highly educated people to an extent that precluded the derivation of poststratification weights that considered education. Educational attainment is positively associated with awareness and use of NFLs, as well as with better understanding of nutrition information [2], as has been found in prior IFPS research [37]. As such, we may have overestimated NFL-related responses in Mexico in spite of our statistical adjustment for education. It is also possible, however that we underestimated policy effects if FoPWLs—which were designed to communicate with low-literacy consumers—were more likely to generate spotlight effects among adults with lower educational attainment. Future research should evaluate this possibility. Our study also involves self-report measures that may be biased. In Mexico, participants were asked a series of questions about the FoPWLs (or the earlier “guideline daily amount” front-of-pack label used in Mexico in 2019) before being asked about NFLs, which may have primed their responses to NFLs relative to United States participants, whose surveys did not include a prior set of questions about front-of-pack labels.

Our difference-in-differences method relies on some assumptions. The parallel trend assumption for the prepolicy period appears to have held for all variables assessed, except for NFL use (Figure 2A–D), which may compromise results from models for that outcome. Furthermore, the stable unit treatment value assumption may not hold given evidence that the Mexican labeling policy spilled over to the United States market through Mexican-oriented stores [49], though contamination is likely to have diluted relative cross-country comparisons. Nevertheless, the difference-in-differences method is likely to minimize any systematic bias around trends within countries that would have affected our assessment of the relative impact of labeling policy implementation. This method helps address concerns about the onset of the COVID-19 pandemic accounting for some of our findings, as consumers may have had fewer opportunities to engage with NFLs in grocery stores due to greater online purchasing; however, for both countries, we would expect that COVID-19 effects would have been stronger for the transition period (November–December 2020) than during the early implementation period (November–December 2021). We further addressed potential concerns about differential COVID-19 effects by estimating early (2019–2021), mid (2019–2022), and late (2019–2023) postpolicy trends in ways that do not consider data from the interval between 2019 and the postpolicy survey year of interest; hence, these trend estimates exclude data from surveys close to the onset of the COVID-19 pandemic. Our findings were also robust to model specification (i.e., consistent when using ordinal regression) and routines to account for missing data. Finally, our study did not have a “control” group without changes to NFLs; nevertheless, the similarity of the updated NFLs across countries, along with the accompanying implementation of FoPWLs in Mexico, provides a meaningful contrast.

Despite these limitations, our study provides evidence that changes to NFLs in the United States did not have a meaningful impact on key labeling outcomes, including awareness, understanding, and use. Desirable changes occurred in Mexico, perhaps due to more substantial changes to its labeling policy. In particular, the simultaneous implementation of updated NFLs and interpretative FoPWLs that go beyond listing of nutritional facts and ingredients by providing broad, yet easy-to-interpret, context for NFL information may be more effective in helping consumers make healthier and more informed food choices.

## Author contributions

The authors’ responsibilities were as follows — JFT: conceptualized the study, wrote the manuscript, and secured funding; VV: wrote the manuscript and helped conceptualize the study; DF: managed data, conducted the analyses, and led the writing of the methods and results sections; ACM and RBA: critically reviewed and edited the manuscript; CMW: managed the project and critically reviewed and edited the manuscript; AJ, RED, LV, JWH, EAF, SB, and DH: helped conceptualize the study, critically reviewed and edited the manuscript, and secured funding; and all authors have read and agreed to the published version of the manuscript.

## Data availability

Disclosure of data online is not covered under the project ethics approvals; however, de-identified participant data from this study will be made available to interested parties upon reasonable request. Interested parties should submit a proposal to the project principal investigator (DH) that he and other key personnel will review for scientific merit. Once a proposal is approved, a data access agreement will need to be signed before the data and relevant protocol information are shared. De-identified survey data will be shared, along with technical reports that include survey protocols, questionnaires, and other relevant details (e.g., poststratification weight development, follow-up rates).

## Declaration of generative AI in scientific writing

AI was not used in any aspect of the writing process for this manuscript.

## Funding

Funding was provided by a Canadian Institutes of Health Research Project Grant (PJT-162167), with additional support from the National Institute of Diabetes and Digestive and Kidney Disorders of the National Institutes of Health (R01 DK128967). The content is solely the responsibility of the authors and does not necessarily represent the official views of the Canadian Institutes for Health Research or the NIH.

## Conflict of interest

The authors report no conflicts of interest.
