# Supplementary material for: Trends in Self-Reported Responses to Nutrition Facts Labels Before and After Nutrition Labeling Policy Implementation: A Comparison of Adults in the United States and Mexico
Source: Curr Dev Nutr. 2026 Feb 22;10(4):107661. doi: 10.1016/j.cdnut.2026.107661 (PMC13068546; doi:10.1016/j.cdnut.2026.107661)
Supplement: Multimedia component 1 [file mmc1.docx]

**Supplementary Analyses**

**Supplementary Table 1. Post- vs. pre-policy trends from ordinal logistic regression models for the salience of nutrition information in stores and awareness, understanding, and use of nutrition facts labels (NFLs): Mexico, the US, and Mexico vs. US, 2018-2023**

|  |  | | OR | | (95% CI) | | p-value | | AOR^1^ | | (95% CI) | | p-value | |
| --- | --- | --- | --- | --- | --- | --- | --- | --- | --- | --- | --- | --- | --- | --- |
| **Ease of finding nutrition info in stores** | |  | |  | |  | |  | |  | |  | |  |
| **Initial policy transition (2019-2020) vs. pre-policy period (2018-2019)** | **Mexico** (within country) | | 1.05 | | (0.89, 1.24) | | 0.573 | | 1.12 | | (0.94, 1.32) | | 0.199 | |
|  | **US** (within country) | | 1.00 | | (0.86, 1.17) | | 0.999 | | 1.01 | | (0.87, 1.19) | | 0.854 | |
|  | **Mexico vs US** | | 1.05 | | (0.83, 1.32) | | 0.681 | | 1.10 | | (0.87, 1.38) | | 0.416 | |
| **Early policy implementation (2019-2021) vs. pre-policy period (2018-2019)** | **Mexico** (within country) | | **1.27** | | **(1.08, 1.50)** | | **0.004** | | **1.27** | | **(1.08, 1.50)** | | **0.004** | |
|  | **US** (within country) | | 0.93 | | (0.80, 1.09) | | 0.397 | | 0.88 | | (0.75, 1.03) | | 0.112 | |
|  | **Mexico vs US** | | **1.36** | | **(1.09, 1.71)** | | **0.008** | | **1.44** | | **(1.15, 1.81)** | | **0.002** | |
| **Mid policy implementation (2019-2022) vs. pre-policy period (2018-2019)** | **Mexico** (within country) | | **1.34** | | **(1.14, 1.58)** | | **<0.001** | | **1.39** | | **(1.18, 1.64)** | | **<0.001** | |
|  | **US** (within country) | | 0.92 | | (0.79, 1.08) | | 0.330 | | 0.96 | | (0.82, 1.12) | | 0.597 | |
|  | **Mexico vs US** | | **1.45** | | **(1.15, 1.83)** | | **0.001** | | **1.45** | | **(1.15, 1.82)** | | **0.002** | |
| **Late policy implementation (2019-2023) vs. pre-policy period (2018-2019)** | **Mexico** (within country) | | **1.43** | | **(1.21, 1.69)** | | **<0.001** | | **1.44** | | **(1.22, 1.70)** | | **<0.001** | |
|  | **US** (within country) | | 0.96 | | (0.82, 1.13) | | 0.636 | | 0.98 | | (0.84, 1.14) | | 0.774 | |
|  | **Mexico vs US** | | **1.48** | | **(1.18, 1.86)** | | **0.001** | | **1.48** | | **(1.17, 1.85)** | | **0.001** | |
| **Extent of awareness of NFLs** | |  | |  | |  | |  | |  | |  | |  |
| **Initial policy transition (2019-2020) vs. pre-policy period (2018-2019)** | **Mexico** (within country) | | 1.08 | | (0.94, 1.26) | | 0.279 | | 1.07 | | (0.92, 1.25) | | 0.351 | |
|  | **US** (within country) | | 0.83 | | (0.69, 1.00) | | 0.050 | | 0.84 | | (0.69, 1.01) | | 0.065 | |
|  | **Mexico vs US** | | **1.31** | | **(1.03, 1.66)** | | **0.027** | | **1.28** | | **(1.01, 1.63)** | | **0.043** | |
| **Early policy implementation (2019-2021) vs. pre-policy period (2018-2019)** | **Mexico** (within country) | | **1.40** | | **(1.21, 1.62)** | | **<0.001** | | **1.39** | | **(1.19, 1.61)** | | **<0.001** | |
|  | **US** (within country) | | 0.84 | | (0.70, 1.02) | | 0.081 | | **0.81** | | **(0.67, 0.98)** | | **0.034** | |
|  | **Mexico vs US** | | **1.66** | | **(1.30, 2.11)** | | **<0.001** | | **1.70** | | **(1.33, 2.17)** | | **<0.001** | |
| **Mid policy implementation (2019-2022) vs. pre-policy period (2018-2019)** | **Mexico** (within country) | | **1.17** | | **(1.01, 1.35)** | | **0.041** | | 1.16 | | (0.99, 1.35) | | 0.06 | |
|  | **US** (within country) | | **0.59** | | **(0.49, 0.72)** | | **<0.001** | | **0.59** | | **(0.49, 0.71)** | | **<0.001** | |
|  | **Mexico vs US** | | **1.97** | | **(1.55, 2.51)** | | **<0.001** | | **1.97** | | **(1.54, 2.51)** | | **<0.001** | |
| **Late policy implementation (2019-2023) vs. pre-policy period (2018-2019)** | **Mexico** (within country) | | 1.12 | | (0.96, 1.30) | | 0.138 | | 1.11 | | (0.95, 1.29) | | 0.184 | |
|  | **US** (within country) | | **0.60** | | **(0.50, 0.73)** | | **<0.001** | | **0.59** | | **(0.49, 0.71)** | | **<0.001** | |
|  | **Mexico vs US** | | **1.86** | | **(1.47, 2.37)** | | **<0.001** | | **1.88** | | **(1.48, 2.40)** | | **<0.001** | |

**Supplementary Table 1. (continued)**

|  |  | | OR | | (95% CI) | | p-value | | AOR^1^ | | (95% CI) | | p-value | |
| --- | --- | --- | --- | --- | --- | --- | --- | --- | --- | --- | --- | --- | --- | --- |
| **Ease of understanding NFLs** | |  | |  | |  | |  | |  | |  | |  |
| **Initial policy transition (2019-2020) vs. pre-policy period (2018-2019)** | **Mexico** (within country) | | 1.07 | | (0.91, 1.26) | | 0.410 | | 1.12 | | (0.96, 1.32) | | 0.154 | |
|  | **US** (within country) | | 0.89 | | (0.76, 1.05) | | 0.154 | | 0.89 | | (0.76, 1.05) | | 0.180 | |
|  | **Mexico vs US** | | 1.20 | | (0.96, 1.51) | | 0.112 | | 1.26 | | (1.00, 1.58) | | 0.051 | |
| **Early policy implementation (2019-2021) vs. pre-policy period (2018-2019)** | **Mexico** (within country) | | **1.44** | | **(1.22, 1.69)** | | **<0.001** | | **1.44** | | **(1.22, 1.69)** | | **<0.001** | |
|  | **US** (within country) | | **0.82** | | **(0.69, 0.96)** | | **0.015** | | **0.76** | | **(0.64, 0.89)** | | **0.001** | |
|  | **Mexico vs US** | | **1.76** | | **(1.40, 2.22)** | | **<0.001** | | **1.90** | | **(1.51, 2.39)** | | **<0.001** | |
| **Mid policy implementation (2019-2022) vs. pre-policy period (2018-2019)** | **Mexico** (within country) | | **1.29** | | **(1.10, 1.52)** | | **0.002** | | **1.32** | | **(1.12, 1.55)** | | **0.001** | |
|  | **US** (within country) | | 0.90 | | (0.76, 1.06) | | 0.214 | | 0.91 | | (0.78, 1.08) | | 0.290 | |
|  | **Mexico vs US** | | **1.44** | | **(1.14, 1.81)** | | **0.002** | | **1.44** | | **(1.15, 1.82)** | | **0.002** | |
| **Late policy implementation (2019-2023) vs. pre-policy period (2018-2019)** | **Mexico** (within country) | | **1.28** | | **(1.09, 1.51)** | | **0.003** | | **1.29** | | **(1.10, 1.52)** | | **0.002** | |
|  | **US** (within country) | | 0.89 | | (0.76, 1.05) | | 0.183 | | 0.89 | | (0.76, 1.05) | | 0.172 | |
|  | **Mexico vs US** | | **1.43** | | **(1.14, 1.80)** | | **0.002** | | **1.45** | | **(1.15, 1.82)** | | **0.002** | |
| **Frequency of using NFLs** | |  | |  | |  | |  | |  | |  | |  |
| **Initial policy transition (2019-2020) vs. pre-policy period (2018-2019)** | **Mexico** (within country) | | **0.83** | | **(0.71, 0.97)** | | **0.021** | | **0.85** | | **(0.73, 1.00)** | | **0.044** | |
|  | **US** (within country) | | 0.97 | | (0.82, 1.14) | | 0.676 | | 0.95 | | (0.80, 1.12) | | 0.522 | |
|  | **Mexico vs US** | | 0.86 | | (0.68, 1.08) | | 0.193 | | 0.90 | | (0.71, 1.13) | | 0.349 | |
| **Early policy implementation (2019-2021) vs. pre-policy period (2018-2019)** | **Mexico** (within country) | | 1.16 | | (0.99, 1.36) | | 0.069 | | 1.14 | | (0.97, 1.33) | | 0.116 | |
|  | **US** (within country) | | 0.87 | | (0.74, 1.03) | | 0.099 | | **0.83** | | **(0.70, 0.97)** | | **0.023** | |
|  | **Mexico vs US** | | **1.33** | | **(1.06, 1.67)** | | **0.014** | | **1.37** | | **(1.09, 1.73)** | | **0.006** | |
| **Mid policy implementation (2019-2022) vs. pre-policy period (2018-2019)** | **Mexico** (within country) | | 1.06 | | (0.91, 1.24) | | 0.474 | | 1.06 | | (0.90, 1.23) | | 0.495 | |
|  | **US** (within country) | | 1.03 | | (0.87, 1.21) | | 0.757 | | 1.03 | | (0.87, 1.22) | | 0.730 | |
|  | **Mexico vs US** | | 1.03 | | (0.82, 1.30) | | 0.790 | | 1.03 | | (0.82, 1.29) | | 0.828 | |
| **Late policy implementation (2019-2023) vs. pre-policy period (2018-2019)** | **Mexico** (within country) | | 1.02 | | (0.87, 1.20) | | 0.776 | | 1.01 | | (0.86, 1.18) | | 0.904 | |
|  | **US** (within country) | | 0.90 | | (0.77, 1.07) | | 0.232 | | 0.89 | | (0.76, 1.05) | | 0.167 | |
|  | **Mexico vs US** | | 1.13 | | (0.90, 1.42) | | 0.289 | | 1.13 | | (0.90, 1.42) | | 0.279 | |

1. Adjusted for sex, age, education, income adequacy, ethnicity, having children at home, shopping role, and weights.

**Supplementary Table 2. Multiple imputation for post- vs. pre-policy trends from linear regression models for the salience of nutrition information in stores and awareness, understanding, and use of nutrition facts labels (NFLs): Mexico, the US, and Mexico vs. US, 2018-2023**

|  |  | | AOR^1^ | (95% CI) | p-value |
| --- | --- | --- | --- | --- | --- |
| **Ease of finding nutrition info in stores** | |  | |  |  |
| **Initial policy transition (2019-2020) vs. pre-policy period (2018-2019)** | **Mexico** (within country) | | 0.045 | (-0.047, 0.138) | 0.338 |
|  | **US** (within country) | | -0.001 | (-0.088, 0.086) | 0.985 |
|  | **Mexico vs US** | | 0.046 | (-0.081, 0.173) | 0.476 |
| **Early policy implementation (2019-2021) vs. pre-policy period (2018-2019)** | **Mexico** (within country) | | **0.123** | **(0.031, 0.215)** | **0.009** |
|  | **US** (within country) | | -0.066 | (-0.153, 0.021) | 0.136 |
|  | **Mexico vs US** | | **0.189** | **(0.062, 0.316)** | **0.004** |
| **Mid policy implementation (2019-2022) vs. pre-policy period (2018-2019)** | **Mexico** (within country) | | **0.162** | **(0.070, 0.254)** | **0.001** |
|  | **US** (within country) | | -0.037 | (-0.125, 0.051) | 0.406 |
|  | **Mexico vs US** | | **0.199** | **(0.072, 0.326)** | **0.002** |
| **Late policy implementation (2019-2023) vs. pre-policy period (2018-2019)** | **Mexico** (within country) | | **0.190** | **(0.097, 0.282)** | **<0.001** |
|  | **US** (within country) | | -0.021 | (-0.108, 0.066) | 0.636 |
|  | **Mexico vs US** | | **0.211** | **(0.084, 0.337)** | **0.001** |
| **Extent of awareness of NFLs** | |  | |  |  |
| **Initial policy transition (2019-2020) vs. pre-policy period (2018-2019)** | **Mexico** (within country) | | 0.032 | (-0.046, 0.111) | 0.423 |
|  | **US** (within country) | | -0.076 | (-0.162, 0.009) | 0.081 |
|  | **Mexico vs US** | | 0.109 | (-0.008, 0.225) | 0.067 |
| **Early policy implementation (2019-2021) vs. pre-policy period (2018-2019)** | **Mexico** (within country) | | **0.138** | **(0.060, 0.217)** | **0.001** |
|  | **US** (within country) | | **-0.095** | **(-0.182, -0.008)** | **0.032** |
|  | **Mexico vs US** | | **0.234** | **(0.116, 0.351)** | **<0.001** |
| **Mid policy implementation (2019-2022) vs. pre-policy period (2018-2019)** | **Mexico** (within country) | | 0.044 | (-0.034, 0.123) | 0.269 |
|  | **US** (within country) | | **-0.255** | **(-0.344, -0.167)** | **<0.001** |
|  | **Mexico vs US** | | **0.300** | **(0.181, 0.419)** | **<0.001** |
| **Late policy implementation (2019-2023) vs. pre-policy period (2018-2019)** | **Mexico** (within country) | | 0.025 | (-0.054, 0.105) | 0.536 |
|  | **US** (within country) | | **-0.247** | **(-0.335, -0.159)** | **<0.001** |
|  | **Mexico vs US** | | **0.272** | **(0.154, 0.391)** | **<0.001** |

**Supplementary Table 2. (continued)**

|  |  | | AOR^1^ | (95% CI) | p-value |
| --- | --- | --- | --- | --- | --- |
| **Ease of understanding NFLs** | |  | |  |  |
| **Initial policy transition (2019-2020) vs. pre-policy period (2018-2019)** | **Mexico** (within country) | | 0.067 | (-0.026, 0.161) | 0.160 |
|  | **US** (within country) | | -0.055 | (-0.141, 0.030) | 0.203 |
|  | **Mexico vs US** | | 0.123 | (-0.004, 0.250) | 0.058 |
| **Early policy implementation (2019-2021) vs. pre-policy period (2018-2019)** | **Mexico** (within country) | | **0.200** | **(0.106, 0.293)** | **<0.001** |
|  | **US** (within country) | | **-0.136** | **(-0.222, -0.050)** | **0.002** |
|  | **Mexico vs US** | | **0.336** | **(0.208, 0.463)** | **<0.001** |
| **Mid policy implementation (2019-2022) vs. pre-policy period (2018-2019)** | **Mexico** (within country) | | **0.164** | **(0.071, 0.257)** | **0.001** |
|  | **US** (within country) | | -0.053 | (-0.139, 0.034) | 0.234 |
|  | **Mexico vs US** | | **0.217** | **(0.090, 0.344)** | **0.001** |
| **Late policy implementation (2019-2023) vs. pre-policy period (2018-2019)** | **Mexico** (within country) | | **0.157** | **(0.064, 0.251)** | **0.001** |
|  | **US** (within country) | | -0.055 | (-0.140, 0.031) | 0.209 |
|  | **Mexico vs US** | | **0.212** | **(0.086, 0.338)** | **0.001** |
| **Frequency of using NFLs** | |  | |  |  |
| **Initial policy transition (2019-2020) vs. pre-policy period (2018-2019)** | **Mexico** (within country) | | **-0.103** | **(-0.205, -0.001)** | **0.048** |
|  | **US** (within country) | | -0.025 | (-0.132, 0.083) | 0.652 |
|  | **Mexico vs US** | | -0.078 | (-0.226, 0.070) | 0.301 |
| **Early policy implementation (2019-2021) vs. pre-policy period (2018-2019)** | **Mexico** (within country) | | 0.098 | (-0.004, 0.201) | 0.060 |
|  | **US** (within country) | | **-0.125** | **(-0.233, -0.017)** | **0.023** |
|  | **Mexico vs US** | | **0.224** | **(0.075, 0.372)** | **0.003** |
| **Mid policy implementation (2019-2022) vs. pre-policy period (2018-2019)** | **Mexico** (within country) | | 0.051 | (-0.050, 0.152) | 0.323 |
|  | **US** (within country) | | 0.013 | (-0.096, 0.122) | 0.816 |
|  | **Mexico vs US** | | 0.038 | (-0.110, 0.186) | 0.615 |
| **Late policy implementation (2019-2023) vs. pre-policy period (2018-2019)** | **Mexico** (within country) | | 0.032 | (-0.070, 0.134) | 0.539 |
|  | **US** (within country) | | -0.069 | (-0.177, 0.039) | 0.211 |
|  | **Mexico vs US** | | 0.101 | (-0.048, 0.250) | 0.183 |

1. Adjusted for sex, age, education, income adequacy, ethnicity, having children at home, shopping role, and weights.
